# White blood cell count and all-cause and cause-specific mortality in the Guangzhou biobank cohort study

**DOI:** 10.1186/s12889-018-6073-6

**Published:** 2018-11-06

**Authors:** Tao Wang, Chao Qiang Jiang, Lin Xu, Wei Sen Zhang, Feng Zhu, Ya Li Jin, G. Neil Thomas, Kar Keung Cheng, Tai Hing Lam

**Affiliations:** 1 School of Public Health, Sun Yat-sen University, 2nd Zhongshan Road, Guangdong Province Guangzhou, China; 2 Guangzhou No.12 Hospital, Guangzhou, 510620 China; 3 School of Public Health, The University of Hong Kong, Hong Kong, China; 4 Institute of Applied Health Research, University of Birmingham, Birmingham, UK

**Keywords:** White blood cell count, Granulocyte count, Cause-specific mortality, All-cause mortality, Prospective cohort study

## Abstract

**Background:**

Several studies have shown positive associations between higher WBC count and deaths from all-causes, CHD, stroke and cancer among occidental populations or developed countries of Asia. No study on the association of WBC count with all-cause and cause-specific mortality in Chinese populations was reported. We studied this using prospective data from a large Chinese cohort.

**Methods:**

We used prospective data from the Guangzhou Biobank Cohort Study (GBCS), a total of 29,925 participants in present study. A Cox proportional hazards regression model was used to estimate the hazard ratios (HR) and 95% confidence interval (CI).

**Results:**

The hazard ratios (HR) for all-cause, CHD, and respiratory disease mortality for the highest decile of WBC count (women > 8.2 × 10^9^/L; men > 8.8 × 10^9^/L) was 1.83 (95% confidence interval (CI) 1.54, 2.17), 3.02 (95% CI 1.84, 4.98) and 2.52 (95% CI 1.49, 4.27), respectively, after adjusting for multiple potential confounders. The associations were similar when deaths during the first 2 years of follow-up were excluded. After further adjusting for pulmonary function, the highest decile of WBC count was associated with 90% higher risk of respiratory disease mortality (HR 1.90, 95% CI 1.08, 3.33). No evidence for an association between higher WBC count and cancer mortality was found. Sub-type analysis showed that only granulocyte count remained significantly predictive of all-cause, CHD, and respiratory disease mortality.

**Conclusions:**

Elevated WBC, specifically granulocyte, count was associated with all-cause, CHD and respiratory mortality in southern Chinese. Further investigation is warranted to clarify whether decreasing inflammation would attenuate WBC count associated mortality.

**Electronic supplementary material:**

The online version of this article (10.1186/s12889-018-6073-6) contains supplementary material, which is available to authorized users.

## Background

In clinical practice, the circulating white blood cell (WBC) count is readily available and widely used as an indicator of systemic inflammation. Numerous studies have shown positive associations between higher WBC count and deaths from all-causes [1–4], coronary heart disease (CHD) [5], stroke [6] and cancer [7]. Most of these studies were conducted in high-income countries of Asia [3, 4] or in the West [1, 2, 5–7]. Ethnic differences in WBC count exist, whereby reference intervals (RIs) for both Americans of Chinese and African origin are lower than those of European and Mexican origin [8]. Moreover, populations with different ethnicities may underlie different patterns of confounding factors (i.e., lifestyle and demographic factors), and an association consistently observed in different settings with different sets of confounders suggests that such association may be real or causal. Given this, and that no study describing the association between WBC count and mortality in Chinese populations was reported, we studied this association for all-cause, CHD, cancer, and respiratory disease mortality using prospective data from the Guangzhou Biobank Cohort Study.

As previous studies suggested an adverse association between pulmonary function and WBC count [9, 10], we hypothesized that this may account for any association between increased WBC count and higher risk of respiratory disease mortality. To examine whether such association, if any, could be attributable to pulmonary function we adjusted for pulmonary function and self-reported pulmonary disease in the analysis of respiratory disease mortality. We also examined the association for sub-types of WBC.

## Methods

### Study subjects

Participants of the Guangzhou Biobank Cohort Study (GBCS) were recruited from September 2003 to January 2008. Details of GBCS were described elsewhere [11]. Briefly, GBCS is a 3-way collaboration among the Guangzhou Number 12 Hospital in Guangzhou, China, the University of Hong Kong in Hong Kong, and the University of Birmingham in the UK. Participants was recruited from the “Guangzhou Health and Happiness Association for the Respectable Elders” (GHHARE), a community social and welfare organization unofficially aligned with the municipal government. Membership is open to Guangzhou permanent residents aged 50+ years for a nominal fee of 4 CNY (≈50 US cents) per month. GHHARE included about 7% of Guangzhou residents in this age group, with branches in all districts of Guangzhou, the capital city of Guangdong Province in southern China.

### Ethical approval

The GBCS was approved by Guangzhou Medical Ethics Committee of the Chinese Medical Association. All participants provided written, informed consent before participation.

### Data collection and variable definition

The baseline examination included an interview about demographic characteristics, lifestyle (smoking and alcohol use), family and personal medical history and assessment of weight, height, blood pressure, fasting plasma glucose, lipids and inflammatory markers. Body mass index was calculated by body weight (kg) divided by height (m) squared. Physical activity was assessed by the Chinese version of International Physical Activity Questionnaire (IPAQ) and was classified into inactive, moderately active and physically active. Reliability and validity of the questionnaire was tested by recalling 200 randomly selected subjects for re-interview. The Kappa values for the following selected questions were: self-reported vascular disease (0.66), smoking (0.96 and 0.88 for the two questions on smoking status), drinking (0.60), education (0.90), and occupation (0.80) [12, 13]. Forced expiratory volume in 1 sec (FEV1) and forced vital capacity (FVC) were measured using spirometry [14]. Fasting blood samples were obtained from all participants at baseline and were analyzed for WBC count (KX-21, SYSMEX, Japan), counts of WBC sub-types, glucose, lipids and other common biochemical parameters.

### Mortality

Information on underlying causes of deaths up to January 2016 was mostly obtained via record linkage with the Guangzhou Center for Disease Control and Prevention (GZCDC). Causes of death were coded according to the 10th revisions of the International Classification of Diseases (ICD) by trained nosologists in each hospital. Cancer types included: breast, liver, stomach, colorectal and lung cancer. When the death certificates were not issued by medical institutions (and hence might have quality issue with the coding), the causes of death were verified by GZCDC as part of their quality assurance programme by cross-checking past medical history and conducting verbal autopsy. In addition, from August 2015 to May 2016, ten verbal autopsy meetings were conducted in the Guangzhou 12th Hospital to further clarify the deaths with unclear causes. A physician panel including 5 chief physicians from various disciplines reviewed all available medical records of the same individuals and assigned in a standard manner a cause of death, with assistance of an epidemiologist for unsettled cases. When participants were confirmed dead but the exact dates of death were not available, the mid-point between the date of recruitment and 31 January 2016 was used (*n* = 6).

### Statistical analysis

Chi-square tests or analysis of variance was used to compare participants’ baseline characteristics by deciles of baseline WBC count. We used Cox proportional hazards models to assess the association of deciles of baseline WBC count with all-cause and cause-specific mortality. We checked the Cox proportional assumption using Schoenfeld residuals by “stphtest” command in Stata. As we found no evidence for the violation of the proportional hazard assumption, the Cox proportional hazards model was used, giving hazard ratios (HRs) and 95% confidence intervals (CI). To enable comparison with previous studies [1, 15], we analyzed WBC count deciles and selected the decile group with the lowest mortality rate as the reference group. We also compared models using different WBC count cut-off points (i.e., tertiles, quartiles or quintiles) in terms of their fitness and found that the model using WBC count deciles had the lowest Akaike information criterion value, indicating a satisfactory fitness. Follow-up time was calculated as days from enrollment to death or the end of follow-up. Covariates included in the main model were age (years), sex (women; men), education (primary or below; secondary; college or above), occupation (manual; non-manual; or others), smoking status (never; former; or current smokers), alcohol use (never; former; or current drinkers), physical activity (inactive; moderate; or active), self-rated health (very poor; poor; good; and very good), body mass index (kg/m^2^), diabetes (yes/no), triglycerides and high-density lipoprotein cholesterol (mmol/l), platelet count (10^9^/l) and haemoglobin (g/l).

Sensitivity analysis was performed by excluding deaths occurred within the first 2 years. For the association of WBC count with respiratory disease mortality, because the association might be confounded by poor lung function, we further adjusted for pulmonary function, as indicated by FVC and FEV1, and self-reported respiratory disease including COPD, chronic bronchitis, emphysema, asthma, tuberculosis, pneumonia. We also tested for an interaction between smoking status and WBC counts using likelihood ratio test to test for the fitness of models with or without the interaction terms. Models with a lower Akaike information criterion (AIC) or Bayesian information criterion (BIC) value indicate better fitness. We found no evidence of interaction between smoking status and WBC count for the risk of all-cause and cause-specific mortality (*P* values for interaction from 0.34 to 0.99). However, as smoking plays a significant role in respiratory disease mortality and mortality due to many other causes, and in other underlying illnesses which can lead to some inflammation, we conducted sensitivity analyses in never or ever- (including former and current) smokers to minimize potential confounding. Furthermore, as a WBC count of less than 4 × 10^9^/L may clinically be an indicator of leucopenia and a WBC count of greater than 10 × 10^9^/L may indicate an elevated inflammatory response, we also conducted sensitivity analyses including participants with a WBC count within the normal range only, as per the internationally recognized criteria for abnormal WBC count (i.e., WBC count < 4 × 10^9^/L or > 10 × 10^9^/L), to minimize the confounding effect due to these underlying diseases. We also did similar analysis for WBC subtypes. All statistical analysis was performed using Stata/IC 14.0.

## Results

Of 30,430 participants at baseline, 391 were excluded because of loss to follow-up with unknown vital status and 202 excluded because of incomplete information on WBC count, giving 29,925 participants (21,648 women and 8277 men) in the present analysis. The mean age at baseline was 62 (standard deviation (SD) 7.1) years. During the average follow-up of 9.3 (SD 1.9) years, 2979 deaths (women 1581 (7.3%); men 1398 (16.9%)) were recorded.

Table 1 shows that the proportion of current smokers increased with increasing deciles of WBC count. Higher BMI, triglycerides, haemoglobin and platelet count were positively, while HDL-cholesterol, FEV1 and FVC was inversely associated with WBC count decile, but current alcohol drinking, physical activity and self-rated health, or age (as a continuous variable) were not.

**Table 1:** Baseline characteristics of participants according to decile of baseline white blood cell count (*n* = 29,925) in Guangzhou Biobank Cohort Study, 2003–2016

	Decile of White Blood Cell Count, × 10^9^/L										
	1st decile (f < 4. 5; m < 4.7) (*n* = 2845)	2nd decile (f 4.5–4.8; m 4.7–5.2) (*n* = 2542)	3rd decile (f 4.9–5.3; m 5.3–5.7) (*n* = 3516)	4th decile (f 5.4–5.6; m 5.8–6.0) (*n* = 2725)	5th decile (f 5.7–6.0; m 6.1–.4) (*n* = 3308)	6th decile (f 6.1–6.3; m 6.5–6.8) (*n* = 2550)	7th decile (f 6.4–6.8; m 6.9–7.2) (*n* = 3293)	8th decile (f 6.9–7.3; m 7.3–7.8) (*n* = 2978)	9th decile (f 7.4–8.2; m 7.9–8.7) (*n* = 3085)	10th decile (f > 8.2; m > 8.8) (*n* = 3083)	*P* for difference
Percentage											
Men	26.3	33.8	23.0	31.6	26.1	30.9	23.1	29.3	27.4	28.3	< 0.001
Education (college or above)	11.5	12.0	10.0	10.1	9.7	8.3	7.0	8.2	7.0	6.2	< 0.001
Occupation (manual)	56.5	57.1	58.2	59.2	59.5	61.1	63.4	63.7	65.1	66.5	< 0.001
Current smokers	5.4	7.6	5.9	8.8	8.8	10.8	8.4	12.7	13.7	18.6	< 0.001
Current alcohol drinkers	23.5	25.1	23.3	26.8	24.5	25.4	22.7	24.9	23.5	23.3	< 0.001
IPAQ Physical activity (active)	49.6	48.5	50.4	51.3	52.6	52.1	52.0	50.6	50.4	50.7	0.006
Self-rated health (good/very good)	81.4	82.6	83.3	83.8	83.3	83.1	83.0	81.4	80.6	79.7	< 0.001
Diabetes	5.9	7.4	8.7	9.7	10.9	11.5	13.5	16.0	18.5	21.1	< 0.001
Mean (SD)											
Age, years	61.3 (7.2)	61.7 (7.1)	61.2 (7)	62 (7.2)	61.8 (7.1)	62 (7)	62.1 (7)	62.5 (7)	62.6 (7.2)	63 (7.1)	< 0.001
Body mass index, kg/m^2^	22.2 (3.1)	22.9 (3.1)	23.3 (3.1)	23.5 (3.1)	23.8 (3.2)	23.9 (3.2)	24.2 (3.2)	24.4 (3.3)	24.5 (3.3)	24.7 (3.5)	< 0.001
Triglycerides, mmol/l	1.2 (0.8)	1.4 (1.0)	1.5 (1.1)	1.6 (1.2)	1.7 (1.3)	1.7 (1.2)	1.8 (1.2)	1.8 (1.4)	2.0 (1.5)	2 (1.5)	< 0.001
HDL-cholesterol, mmol/l	1.8 (0.4)	1.7 (0.4)	1.7 (0.4)	1.6 (0.4)	1.7 (0.4)	1.6 (0.4)	1.6 (0.4)	1.6 (0.4)	1.6 (0.4)	1.6 (0.4)	< 0.001
LDL-cholesterol, mmol/l	3.15 (0.69)	3.24 (0.72)	3.27 (0.70)	3.24 (0.69)	3.28 (0.71)	3.29 (0.71)	3.27 (0.70)	3.29 (0.70)	3.28 (0.70)	3.30 (0.73)	< 0.001
Haemoglobin, g/l	131.3 (13.7)	134.9 (13.6)	134.3 (12.9)	136.3 (13)	136.5 (12.9)	137.4 (13.4)	137.1 (12.9)	138.1 (13.5)	138.5 (13.8)	139.8 (13.8)	< 0.001
Platelet count, 10^9^/l	190.7 (51.3)	204.7 (50.1)	215 (62.7)	218.7 (48.8)	224.8 (50.9)	230 (52.2)	234.5 (57.8)	239.5 (55.8)	246.1 (55.4)	262.8 (77.8)	< 0.001
Waist circumference, cm	82.67 (17.2)	80.30 (9.14)	81.23 (18.60)	81.85 (9.10)	82.60 (17.3)	82.95 (8.98)	83.79 (17.56)	84.00 (9.09)	84.13 (9.11)	84.82 (8.90)	0.32
FEV1, L	1.96 (0.52)	2 (1.39)	1.96 (3.65)	1.92 (0.54)	1.93 (1.67)	1.91 (0.54)	1.86 (1.24)	1.9 (3.67)	1.8 (0.52)	1.75 (0.55)	< 0.001
FVC, L	2.52 (0.71)	2.55 (0.71)	2.44 (0.63)	2.47 (0.65)	2.42 (0.62)	2.45 (0.66)	2.36 (0.66)	2.36 (0.67)	2.33 (0.74)	2.28 (0.68)	< 0.001

For both all-cause and CHD mortality, the 4th decile group (i.e., WBC count of 5.4–5.6 × 10^9^/L in women and 5.8–6.0 × 10^9^/L in men) had the lowest mortality rate. Hence, the 4th decile group was used as the reference. Table 2 shows that greater WBC count was associated with both all-cause and CHD mortality in multivariable adjusted models, and the associations were slightly attenuated but remained statistically significant when the deaths in the first 2 years of follow-up were excluded. Relative to the 4th decile, the adjusted HRs for both all-cause and CHD mortality increased substantially in the highest decile (women > 8.2 × 10^9^/L; men > 8.8 × 10^9^/L; HR 1.83 (95% CI 1.54, 2.17) and 3.02 (1.84, 4.98), respectively).

**Table 2:** Association between baseline white blood cell count and total mortality and coronary heart disease (CHD) mortality in the Guangzhou Biobank Cohort Study, 2003–2016

Decile of WBC count, ×10^9^/L (range)	Age, sex-adjusted results (*n* = 29,925)		Multivariable adjusted^a^ (*n* = 29,925)		Further excluding deaths within 2 years^a^ (*n* = 29,672)	
	HR (95% CI)	*P* value	HR (95% CI)	*P* value	HR (95% CI)	*P* value
Total mortality (no. of deaths = 2997)						
1st decile (f < 4.5; m < 4.7)	1.36 (1.14, 1.63)	0.001	1.31 (1.09, 1.58)	0.005	1.20 (0.99, 1.46)	0.07
2nd decile (f 4.5–4.8; m 4.7–5.2)	1.32 (1.10, 1.58)	0.003	1.33 (1.10, 1.60)	0.003	1.26 (1.04, 1.53)	0.02
3rd decile (f 4.9–5.3; m 5.3–5.7)	1.06 (0.88, 1.28)	0.51	1.05 (0.87, 1.27)	0.63	0.97 (0.80, 1.18)	0.77
4th decile (f 5.4–5.6; m 5.8–6.0)	Reference		Reference		Reference	
5th decile (f 5.7–6.0; m 6.1–.4)	1.30 (1.09, 1.54)	0.004	1.29 (1.08, 1.55)	0.005	1.21 (1.00, 1.46)	0.04
6th decile (f 6.1–6.3; m 6.5–6.8)	1.13 (0.94, 1.37)	0.20	1.14 (0.94, 1.38)	0.20	1.09 (0.90, 1.34)	0.38
7th decile (f 6.4–6.8; m 6.9–7.2)	1.36 (1.14, 1.62)	0.001	1.36 (1.13, 1.63)	0.001	1.28 (1.06, 1.53)	0.009
8th decile (f 6.9–7.3; m 7.3–7.8)	1.56 (1.31, 1.85)	< 0.001	1.50 (1.26, 1.79)	< 0.001	1.41 (1.17, 1.69)	< 0.001
9th decile (f 7.4–8.2; m 7.9–8.7)	1.68 (1.42, 1.99)	< 0.001	1.58 (1.32, 1.88)	< 0.001	1.48 (1.23, 1.77)	< 0.001
10th decile (f > 8.2; m > 8.8)	1.96 (1.66, 2.31)	< 0.001	1.83 (1.54, 2.17)	< 0.001	1.72 (1.44, 2.05)	< 0.001
CHD mortality (no. of deaths = 459)^b^						
1st decile (f < 4.5; m < 4.7)	1.48 (0.85, 2.57)	0.16	1.54 (0.87, 2.71)	0.14	1.48 (0.84, 2.61)	0.18
2nd decile (f 4.5–4.8; m 4.7–5.2)	1.20 (0.67, 2.15)	0.55	1.28 (0.70, 2.31)	0.42	1.22 (0.67, 2.22)	0.52
3rd decile (f 4.9–5.3; m 5.3–5.7)	1.47 (0.86, 2.52)	0.16	1.40 (0.80, 2.44)	0.24	1.40 (0.80, 2.43)	0.24
4th decile (f 5.4–5.6; m 5.8–6.0)	Reference		Reference		Reference	
5th decile (f 5.7–6.0; m 6.1–.4)	1.51 (0.89, 2.57)	0.13	1.48 (0.86, 2.55)	0.16	1.34 (0.77, 2.34)	0.30
6th decile (f 6.1–6.3; m 6.5–6.8)	1.58 (0.91, 2.74)	0.11	1.58 (0.90, 2.78)	0.11	1.60 (0.91, 2.81)	0.10
7th decile (f 6.4–6.8; m 6.9–7.2)	1.85 (1.11, 3.10)	0.02	1.78 (1.05, 3.02)	0.03	1.74 (1.02, 2.95)	0.04
8th decile (f 6.9–7.3; m 7.3–7.8)	2.40 (1.46, 3.95)	0.001	2.27 (1.36, 3.78)	0.002	2.13 (1.27, 3.56)	0.004
9th decile (f 7.4–8.2; m 7.9–8.7)	1.74 (1.03, 2.93)	0.04	1.62 (0.95, 2.78)	0.08	1.56 (0.90, 2.68)	0.11
10th decile (f > 8.2; m > 8.8)	3.45 (2.15, 5.55)	< 0.001	3.02 (1.84, 4.98)	< 0.001	2.86 (1.73, 4.73)	< 0.001

^a^ adjusted for age, sex, education, occupation, smoking status, alcohol use, physical activity, self-rated health, body mass index, diabetes, triglycerides, high-density lipoprotein cholesterol, platelet count and haemoglobin

^b^ 1528 participant with vascular disease at baseline including coronary heart disease, stroke, angina, myocardial infarction and peripheral vascular disease were excluded

For cancer mortality and respiratory disease mortality, the lowest mortality rate was observed in the 3rd decile group (i.e., WBC count of 4.9–5.3 × 10^9^/L in women and 5.3–5.7 × 10^9^/L in men), which was used as the reference group in the subsequent analyses. Table 3 shows that, relative to the 3rd decile group, participants in the lower decile (1st or 2nd) showed a higher risk for cancer mortality, and the HR was statistically significant for the 2nd decile (HR 1.54, 95% CI 1.17, 2.03). Greater WBC count showed no association with cancer mortality. We found positive associations between higher deciles of WBC count and respiratory disease mortality in the multivariable adjusted models, and the associations were unchanged after excluding deaths within the first 2 years of follow-up (Table 3). After further adjustment for pulmonary function and respiratory disease history including COPD, chronic bronchitis, emphysema, asthma, tuberculosis, pneumonia, the associations were attenuated slightly but remained statistically significant (HR 1.90, 95% CI 1.08, 3.33). After excluding current and former smokers, the results were similar (Table 4).

**Table 3:** Association between the baseline white blood cell count and cancer and respiratory disease mortality in the Guangzhou Biobank Cohort Study, 2003–2016

Decile of WBC count, ×10^9^/L (range)	Age, sex-adjusted results (*n* = 29,925)		Multivariable adjusted^a^ (*n* = 29,925)		Further excluding deaths within 2 years^a^ (*n* = 29,672)	
	HR (95% CI)	*P* value	HR (95% CI)	*P* value	HR (95% CI)	*P* value
Cancer mortality (no. of deaths = 1153)						
1st decile (f < 4. 5; m < 4.7)	1.33 (1.01, 1.75)	0.04	1.33 (1.00, 1.77)	0.05	1.29 (0.95, 1.75)	0.10
2nd decile (f 4.5–4.8; m 4.7–5.2)	1.50 (1.15, 1.97)	0.003	1.54 (1.17, 2.03)	0.002	1.52 (1.14, 2.03)	0.005
3rd decile (f 4.9–5.3; m 5.3–5.7)	Reference		Reference		Reference	
4th decile (f 5.4–5.6; m 5.8–6.0)	1.05 (0.79, 1.40)	0.74	1.05 (0.78, 1.41)	0.75	1.10 (0.81, 1.50)	0.52
5th decile (f 5.7–6.0; m 6.1–.4)	1.23 (0.94, 1.61)	0.13	1.20 (0.91, 1.58)	0.20	1.19 (0.89, 1.58)	0.25
6th decile (f 6.1–6.3; m 6.5–6.8)	1.02 (0.76, 1.37)	0.91	1.03 (0.76, 1.39)	0.86	0.99 (0.72, 1.36)	0.94
7th decile (f 6.4–6.8; m 6.9–7.2)	1.15 (0.88, 1.52)	0.30	1.14 (0.86, 1.51)	0.35	1.11 (0.83, 1.49)	0.49
8th decile (f 6.9–7.3; m 7.3–7.8)	1.37 (1.05, 1.78)	0.02	1.30 (0.99, 1.71)	0.06	1.28 (0.96, 1.70)	0.10
9th decile (f 7.4–8.2; m 7.9–8.7)	1.36 (1.04, 1.77)	0.02	1.24 (0.94, 1.63)	0.14	1.25 (0.94, 1.67)	0.13
10th decile (f > 8.2; m > 8.8)	1.27 (0.97, 1.66)	0.09	1.13 (0.85, 1.50)	0.41	1.13 (0.84, 1.52)	0.42
Respiratory disease mortality (no. of deaths = 325)						
1st decile (f < 4. 5; m < 4.7)	1.23 (0.69, 2.19)	0.49	1.09 (0.59, 1.99)	0.78	1.16 (0.62, 2.17)	0.64
2nd decile (f 4.5–4.8; m 4.7–5.2)	1.19 (0.66, 2.14)	0.55	1.23 (0.68, 2.22)	0.50	1.30 (0.70, 2.40)	0.41
3rd decile (f 4.9–5.3; m 5.3–5.7)	Reference		Reference		Reference	
4th decile (f 5.4–5.6; m 5.8–6.0)	1.36 (0.78, 2.37)	0.28	1.41 (0.80, 2.50)	0.24	1.55 (0.86, 2.79)	0.15
5th decile (f 5.7–6.0; m 6.1–.4)	1.44 (0.84, 2.47)	0.19	1.52 (0.87, 2.64)	0.14	1.50 (0.83, 2.68)	0.18
6th decile (f 6.1–6.3; m 6.5–6.8)	1.41 (0.80, 2.49)	0.23	1.47 (0.82, 2.65)	0.20	1.61 (0.88, 2.95)	0.12
7th decile (f 6.4–6.8; m 6.9–7.2)	1.47 (0.85, 2.52)	0.17	1.55 (0.89, 2.72)	0.12	1.53 (0.85, 2.76)	0.16
8th decile (f 6.9–7.3; m 7.3–7.8)	2.07 (1.24, 3.45)	0.005	2.19 (1.29, 3.72)	0.004	2.28 (1.31, 3.95)	0.003
9th decile (f 7.4–8.2; m 7.9–8.7)	1.99 (1.19, 3.34)	0.01	2.25 (1.32, 3.85)	< 0.001	2.26 (1.29, 3.95)	< 0.001
10th decile (f > 8.2; m > 8.8)	2.32 (1.41, 3.84)	0.001	2.52 (1.49, 4.27)	0.001	2.51 (1.45, 4.35)	0.001

^a^ adjusted for age, sex, education, occupation, smoking status, alcohol use, physical activity, self-rated health, body mass index, diabetes, triglycerides, high-density lipoprotein cholesterol, platelet count and haemoglobin

**Table 4:** Association between the baseline white blood cell count and respiratory disease mortality in the Guangzhou Biobank Cohort Study, 2003–2016, excluding deaths within 2 years

Decile of WBC count, ×10^9^/L (range)	Multivariable model^a^ (*n* = 29,672)		Multivariable model in non-smokers ^a^ (*n* = 23,958)	
	HR (95% CI)	*P* value	HR (95% CI)	*P* value
1st decile (f < 4. 5; m < 4.7)	1.28 (0.68, 2.41)	0.44	1.20 (0.52, 2.80)	0.67
2nd decile (f 4.5–4.8; m 4.7–5.2)	1.21 (0.65, 2.25)	0.56	1.07 (0.45, 2.54)	0.88
3rd decile (f 4.9–5.3; m 5.3–5.7)	Reference		Reference	
4th decile (f 5.4–5.6; m 5.8–6.0)	1.50 (0.83, 2.72)	0.18	1.51 (0.69, 3.31)	0.31
5th decile (f 5.7–6.0; m 6.1–.4)	1.39 (0.78, 2.51)	0.27	1.45 (0.67, 3.14)	0.35
6th decile (f 6.1–6.3; m 6.5–6.8)	1.49 (0.81, 2.73)	0.20	1.74 (0.79, 3.85)	0.17
7th decile (f 6.4–6.8; m 6.9–7.2)	1.36 (0.75, 2.46)	0.31	1.39 (0.64, 3.03)	0.40
8th decile (f 6.9–7.3; m 7.3–7.8)	1.89 (1.08, 3.31)	0.03	1.91 (0.90, 4.04)	0.09
9th decile (f 7.4–8.2; m 7.9–8.7)	1.99 (1.13, 3.48)	0.02	2.13 (1.01, 4.48)	0.05
10th decile (f > 8.2; m > 8.8)	1.90 (1.08, 3.33)	0.03	1.79 (0.83, 3.84)	0.14

^a^: adjusted for age, sex, education, occupation, smoking status, alcohol use, physical activity, self-rated health, body mass index, diabetes, triglycerides, high-density lipoprotein cholesterol, platelet count, haemoglobin, force vital capacity (FVC), forced expiratory volume in 1 s (FEV1), and self-reported respiratory disease (COPD, chronic bronchitis, emphysema, asthma, tuberculosis, pneumonia)

Sensitivity analysis showed that, after excluding participants with abnormal WBC count, or restricting data analysis to current smokers only, the results showing that greater WBC count was associated with increased respiratory disease mortality were similar (Additional file 1: Tables S1-S4). Another sensitivity analysis examining the association of subtypes of WBC (i.e., granulocyte or lymphocyte count) with mortality showed that participants with higher granulocyte count (> 5.44 × 10^9^/L) were associated with 45%, 69% and 1.6-fold higher risk of mortality from all-cause, CHD and respiratory disease, respectively, but not associated with cancer mortality (Additional file 1: Tables S5-S6). However, no association between lymphocyte count and mortality was found (Additional file 1: Tables S7-S8).

## Discussion

In this understudied population, high WBC count was robustly associated with all-cause, CHD and respiratory disease mortality after adjusting for potential confounders. The significant association between high WBC count and respiratory disease mortality supports our hypothesis that an increased in WBC count predicts a higher risk of respiratory disease mortality, independently of smoking and pulmonary function.

WBC count is a standardized and accurate measurement that has been used in epidemiologic studies as a predictor of mortality from all- and specific causes in the general population [1, 3, 4, 16, 17], or a predictor of specific outcomes in hospital-based studies of patients [18–21]. Our results on all-cause and CHD mortality are consistent with those from other settings [1, 3, 4]. The association of WBC count with cardiovascular risk or mortality has been well described in studies that have focused on western or economically-developed Asian populations [2–5, 22]. Such association persists after adjustment for multiple cardiovascular risk factors including smoking, an important confounder of the WBC-mortality association [23]. We found no evidence for an interaction between smoking and WBC count, which is in agreement with previous studies [23, 24], supporting an independent association between WBC count and risk of CHD in smokers and in non-smokers. Elevated WBC count may affect CHD through a number of possible pathologic mechanisms, such as altering endothelial function [25], attenuating nitric oxide and prostacyclin production [26] and consequently reducing the vasodilatory, antithrombotic and anti-atherogenic properties of the vascular endothelium [27, 28]. Increased adherence of stimulated WBC to the vascular endothelium promotes capillary leukocytosis and subsequent increased vascular resistance [29].

Results of previous prospective cohort studies on the association of WBC count with cancer mortality have been less consistent. We found only 6 studies [1, 3, 7, 30–32], and of them, three showed a positive association [7, 31, 32], two showed no association [1, 3] and one showed an inverse association [7]. In a subset of participants in the Multiple Risk Factor Intervention Trial (MRFIT), elevated WBC count was associated with a higher risk of cancer death [31]. A prospective study conducted in postmenopausal women also found the evidence of higher WBC counts associated with higher risk of breast, lung and overall cancer mortality [32]. The other study from Australia also showed a higher risk of cancer mortality in those with high (4th quartile) versus low WBC count (1st quartile group) [7]. However, a large Korean study found no association between WBC count and cancer mortality, although, as expected, it found a significant and robust association with CHD mortality [3]. In a more recent study from the Women’s Health Initiative (WHI), the association of WBC count with cancer mortality was weaker than those of all-cause or CHD mortality, and was attenuated toward the null when women with comorbidity and deaths within the first 6 years of follow-up were excluded [1]. Our results also indicate a possible association between lower WBC count and cancer mortality, which was observed in the WHI study and a Taiwan study [30]. Within the normal range, a relatively low WBC count may be an indicator of low immune response which could also increase the risk of cancer mortality, although the underlying mechanisms warrant further investigation. We did not find any evidence supporting an association of higher WBC count with cancer mortality. As some treatment for cancer may lower WBC counts in clinical practice and cancer patients may be more vulnerable to opportunistic pathogens, both are often associated with a higher risk of infection that requires treatment with antibiotics and may affect WBC counts subsequently. However, our sensitivity analysis excluding participants with self-reported cancer history showed similar results.

Our study showed that elevated WBC count was significantly associated with a higher risk of respiratory mortality. Previous large cohort studies suggested that such an association could be due to confounding from smoking [33], i.e., smoking leads to both an elevated WBC count and a higher risk of respiratory mortality [33–35]. Using baseline data of the GBCS, we also found that relative to never smokers, current smokers were associated with a greater WBC count and demonstrated a dose response relationship [36]. However, in the present analysis, the association was still observed in our never smokers, suggesting that smoking was unlikely to account for the findings. One possible mechanism is that systemic inflammation-induced endothelial dysfunction may lead to pulmonary vascular filtration and lung tissue injury [9]. In addition, neutrophils, one type of granulocytes, secrete serine proteases including neutrophil elastase, cathepsin G and proteinase-3, as well as MMP-8 and MMP-9, which may lead to destruction of the alveolar tissue and increase the risk of lung disease [37]. The exact mechanisms underlying the association of WBC count with cause-specific mortality require investigation.

For the association with CVD/CHD, we found six studies [38–43] showing a positive association with granulocyte count, and of them, four showed no association with lymphocyte or monocyte counts [38, 39, 41, 42], and one showed a positive association with monocyte count [40]. A meta-analysis showed that people in the highest, versus those in the lowest, granulocyte quartile had a risk ratio of 1.32 (95% CI 1.15–1.51) for CHD [44]. Our study is in accordance with this meta-analysis showing that participants with higher granulocyte count were associated with a 69% higher risk of CHD mortality. Our results suggest that the increased risk related to WBC count might be partly due to the granulocyte count. Further studies exploring the differentiation, production and function of granulocytes and the related health effects are warranted. Although in our present study, the number of deaths was not sufficient to support more analyses for the subtypes of cardiovascular events, we would follow up all participants to enable more in-depth analysis.

The strengths of this study included the large sample size and comprehensive measurement and adjustment of potential confounding factors. However, there were some limitations. First, as noted above, the number of deaths from cause-specific mortality (especially CHD or respiratory disease) was relatively small. More detailed analyses of specific types of cardiovascular or respiratory disease could not be performed. Moreover, participants in the lower decile groups appeared to have a higher risk of death from all-cause, CHD and respiratory disease, although the results were not statistically significant. As the number of deaths in the lower decile groups were small, further studies are warranted to clarify. Second, as baseline WBC count was measured only at one-time point, the WBC counts might not accurately indicate a lifelong exposure. Repeated WBC measurements may provide a more reliable measurement of WBC count and minimize potential misclassification of exposure. However, in a recent study in women, similar results were observed using a single baseline measurement and using the average of the measurements at baseline and at the third year [1]. Thus, the lack of repeated measurement in the current study could not have influenced the risk estimation substantially. Third, our results based on prospective observational data cannot confirm a causal relation between WBC count and deaths due to CHD or respiratory disease. But as a predictive biomarker, the results from our study are informative and complementary to the literature. Fourth, although we have adjusted for a range of potential confounding factors, elevated WBC count may reflect an inflammatory response due to unknown causes (i.e., underlying chronic diseases or specific exposures that would increase WBC count and mortality at the same time). An alternative newly developed method, Mendelian randomization, might provide a unique opportunity to investigate the direct association, if any, between WBC count and CHD or respiratory disease. Fifth, women were oversampled in this study, as other population-based elderly cohorts. However, we tested for sex interaction and found no evidence for interaction (*P* values for interaction ranged 0.33 to 0.91), indicating the association between WBC count and the risk of all-cause and cause-specific mortality did not vary by sex. Moreover, sex was also adjusted in all models to minimize its potential confounding effect. Thus, the unbalanced sex ratio might not be a major concern in the current study. Finally, as all participants in the GBCS were older Chinese from southern China, generalizability may be a concern, although within sex and age group, the participants had fairly similar prevalence of major chronic diseases (i.e., diabetes and hypertension) to nationally representative samples of urban Chinese [11].

## Conclusions

In conclusion, our results provide robust prospective evidence supporting that WBC and specifically granulocyte count is associated with the risk of all-cause, CHD and respiratory mortality. Further investigation is warranted to clarify whether decreases in inflammation would have effects on WBC count associated mortality.

## Additional file

Additional file 1: **Table S1.** Association between the baseline white blood cell count within normal range (4 × 10^9^/L < WBC count < 10 × 10^9^/L) and total mortality and coronary heart disease (CHD) mortality in the Guangzhou Biobank Cohort Study, 2003–2016; **Table S2.** Association between the baseline white blood cell count within normal range (4 × 10^9^/L < WBC count < 10 × 10^9^/L) and cancer and respiratory disease mortality in the Guangzhou Biobank Cohort Study, 2003–2016; **Table S3.** Association between the baseline white blood cell count within normal range (4 × 10^9^/L < WBC count < 10 × 10^9^/L) and respiratory disease mortality in the Guangzhou Biobank Cohort Study, 2003–2016, excluding deaths within 2 years; **Table S4.** Association between the baseline white blood cell count and respiratory disease mortality in current smokers in the Guangzhou Biobank Cohort Study, 2003–2016; **Table S5**. Association between the baseline granulocyte count and total mortality and coronary heart disease (CHD) mortality in the Guangzhou Biobank Cohort Study, 2003–2016; **Table S6.** Association between the baseline granulocyte count and cancer and respiratory disease mortality in the Guangzhou Biobank Cohort Study, 2003–2016; **Table S7.** Association between the baseline lymphocyte count and total mortality and coronary heart disease (CHD) mortality in the Guangzhou Biobank Cohort Study, 2003–2016; **Table S8.** Association between the baseline lymphocyte count and cancer and respiratory disease mortality in the Guangzhou Biobank Cohort Study, 2003–2016. (DOCX 41 kb)

## Abbreviations

CHD: Coronary heart disease
CI: Confidence interval
FEV1: Forced expiratory volume in one second
FVC: Forced vital capacity
GBCS: The Guangzhou Biobank Cohort Study
HRs: Hazard ratios
IPAQ: International Physical Activity Questionnaire
SD: Standard deviation
WBC: White blood cell
