# Supplementary material for: White blood cell count and all-cause and cause-specific mortality in the Guangzhou biobank cohort study
Source: BMC Public Health. 2018 Nov 6;18:1232. doi: 10.1186/s12889-018-6073-6 (PMC6219250; doi:10.1186/s12889-018-6073-6)
Supplement: Supplementary file 1 — Table S1. Association between the baseline white blood cell count within normal range (4 × 10^9^/L < WBC count < 10 × 109/L) and total mortality and coronary heart disease (CHD) mortality in the Guangzhou Biobank Cohort Study, 2003–2016; Table S2. Association between the baseline white blood cell count within normal range (4 × 10^9^/L < WBC count < 10 × 109/L) and cancer and respiratory disease mortality in the Guangzhou Biobank Cohort Study, 2003–2016; Table S3. Association between the baseline white blood cell count within normal range (4 × 10^9^/L < WBC count < 10 × 109/L) and respiratory disease mortality in the Guangzhou Biobank Cohort Study, 2003–2016, excluding deaths within 2 years; Table S4. Association between the baseline white blood cell count and respiratory disease mortality in current smokers in the Guangzhou Biobank Cohort Study, 2003–2016; Table S5. Association between the baseline granulocyte count and total mortality and coronary heart disease (CHD) mortality in the Guangzhou Biobank Cohort Study, 2003–2016; Table S6. Association between the baseline granulocyte count and cancer and respiratory disease mortality in the Guangzhou Biobank Cohort Study, 2003–2016; Table S7. Association between the baseline lymphocyte count and total mortality and coronary heart disease (CHD) mortality in the Guangzhou Biobank Cohort Study, 2003–2016; Table S8. Association between the baseline lymphocyte count and cancer and respiratory disease mortality in the Guangzhou Biobank Cohort Study, 2003–2016. (DOCX 41 kb) [file 12889_2018_6073_MOESM1_ESM.docx]

Table S1. Association between the baseline white blood cell count within normal range (4×10^9^/L < WBC count < 10×10^9^/L) and total mortality and coronary heart disease (CHD) mortality in the Guangzhou Biobank Cohort Study, 2003-2016

| **Decile of WBC count, ×10^9^/L (range)** | **Age, sex-adjusted results**  **(n=27,886)** | | **Multivariable adjusted ^a^**  **(n=27,886)** | | **Further excluding deaths**  **within 2 years ^a^ (n=27,668)** | |
| --- | --- | --- | --- | --- | --- | --- |
|  | HR (95% CI) | *P* value | HR (95% CI) | *P* value | HR (95% CI) | *P* value |
| **Total mortality (no. of deaths=2,702)** | | | | | | |
| 1^st^ decile (f 4.0-4.5; m 4.0-4.7) | 1.21 (0.98, 1.50) | 0.07 | 1.17 (0.93, 1.45) | 0.17 | 1.09 (0.87, 1.37) | 0.46 |
| 2^nd^ decile (f 4.5-4.8; m 4.7-5.2) | 1.32 (1.10, 1.59) | 0.003 | 1.33 (1.10, 1.61) | 0.003 | 1.26 (1.04, 1.53) | 0.02 |
| 3^rd^ decile (f 4.9-5.3; m 5.3-5.7) | 1.06 (0.88, 1.28) | 0.52 | 1.05 (0.87, 1.27) | 0.62 | 0.97 (0.80, 1.18) | 0.76 |
| 4^th^ decile (f 5.4-5.6; m 5.8-6.0) | Reference |  | Reference |  | Reference |  |
| 5^th^ decile (f 5.7-6.0; m 6.1-.4) | 1.29 (1.09, 1.54) | 0.004 | 1.29 (1.08, 1.54) | 0.006 | 1.21 (1.00, 1.45) | 0.05 |
| 6^th^ decile (f 6.1-6.3; m 6.5-6.8) | 1.13 (0.94, 1.37) | 0.20 | 1.14 (0.93, 1.38) | 0.21 | 1.09 (0.89, 1.33) | 0.39 |
| 7^th^ decile (f 6.4-6.8; m 6.9-7.2) | 1.36 (1.14, 1.62) | 0.001 | 1.35 (1.13, 1.62) | 0.001 | 1.27 (1.06, 1.53) | 0.01 |
| 8^th^ decile (f 6.9-7.3; m 7.3-7.8) | 1.56 (1.31, 1.85) | <0.001 | 1.49 (1.25, 1.78) | <0.001 | 1.40 (1.17, 1.68) | <0.001 |
| 9^th^ decile (f 7.4-8.2; m 7.9-8.7) | 1.68 (1.42, 1.99) | <0.001 | 1.57 (1.31, 1.87) | <0.001 | 1.47 (1.23, 1.76) | <0.001 |
| 10^th^ decile (f 8.2-10; m 8.8-10) | 1.72 (1.44, 2.06) | <0.001 | 1.60 (1.33, 1.93) | <0.001 | 1.52 (1.25, 1.84) | <0.001 |
| **CHD mortality (no. of deaths=422)^b^** | | | | | | |
| 1^st^ decile (f 4.0-4.5; m 4.0-4.7) | 1.46 (0.78, 2.72) | 0.24 | 1.52 (0.80, 2.87) | 0.20 | 1.52 (0.81, 2.87) | 0.20 |
| 2^nd^ decile (f 4.5-4.8; m 4.7-5.2) | 1.20 (0.67, 2.15) | 0.55 | 1.27 (0.70, 2.30) | 0.43 | 1.21 (0.67, 2.22) | 0.53 |
| 3^rd^ decile (f 4.9-5.3; m 5.3-5.7) | 1.46 (0.85, 2.50) | 0.17 | 1.39 (0.80, 2.43) | 0.25 | 1.39 (0.79, 2.42) | 0.25 |
| 4^th^ decile (f 5.4-5.6; m 5.8-6.0) | Reference |  | Reference |  | Reference |  |
| 5^th^ decile (f 5.7-6.0; m 6.1-.4) | 1.51 (0.88, 2.56) | 0.13 | 1.48 (0.86, 2.56) | 0.16 | 1.34 (0.77, 2.35) | 0.30 |
| 6^th^ decile (f 6.1-6.3; m 6.5-6.8) | 1.57 (0.90, 2.73) | 0.11 | 1.59 (0.91, 2.80) | 0.11 | 1.60 (0.91, 2.82) | 0.10 |
| 7^th^ decile (f 6.4-6.8; m 6.9-7.2) | 1.84 (1.10, 3.07) | 0.02 | 1.78 (1.05, 3.02) | 0.03 | 1.73 (1.02, 2.94) | 0.04 |
| 8^th^ decile (f 6.9-7.3; m 7.3-7.8) | 2.39 (1.45, 3.93) | 0.001 | 2.28 (1.37, 3.80) | 0.002 | 2.13 (1.27, 3.57) | 0.004 |
| 9^th^ decile (f 7.4-8.2; m 7.9-8.7) | 1.72 (1.02, 2.91) | 0.04 | 1.62 (0.95, 2.79) | 0.08 | 1.55 (0.90, 2.68) | 0.11 |
| 10^th^ decile (f 8.2-10; m 8.8-10) | 3.37 (2.06, 5.51) | <0.001 | 3.02 (1.80, 5.07) | <0.001 | 2.84 (1.68, 4.79) | <0.001 |

^a^: adjusted for age, sex, education, occupation, smoking status, alcohol use, physical activity, self-rated health, body mass index, diabetes, triglycerides, high-density lipoprotein cholesterol, platelet count and haemoglobin.

^b^: 1528 participant with vascular disease at baseline including coronary heart disease, stroke, angina, myocardial infarction and peripheral vascular disease were excluded.

Table S2. Association between the baseline white blood cell count within normal range (4×10^9^/L < WBC count < 10×10^9^/L) and cancer and respiratory disease mortality in the Guangzhou Biobank Cohort Study, 2003-2016

| **Decile of WBC count, ×10^9^/L (range)** | **Age, sex-adjusted results**  **(n=27,886)** | | **Multivariable adjusted ^a^**  **(n=27,886)** | | **Further excluding deaths**  **within 2 years ^a^ (n=27,668)** | |
| --- | --- | --- | --- | --- | --- | --- |
|  | HR (95% CI) | *P* value | HR (95% CI) | *P* value | HR (95% CI) | *P* value |
| **Cancer mortality (no. of deaths=1,036)** |  |  |  |  |  |  |
| 1^st^ decile (f 4.0-4.5; m 4.0-4.7) | 1.17 (0.84, 1.63) | 0.35 | 1.14 (0.81, 1.60) | 0.47 | 1.16 (0.81, 1.66) | 0.41 |
| 2^nd^ decile (f 4.5-4.8; m 4.7-5.2) | 1.51 (1.15, 1.98) | 0.003 | 1.55 (1.17, 2.04) | 0.002 | 1.53 (1.14, 2.04) | 0.004 |
| 3^rd^ decile (f 4.9-5.3; m 5.3-5.7) | Reference |  | Reference |  | Reference |  |
| 4^th^ decile (f 5.4-5.6; m 5.8-6.0) | 1.05 (0.79, 1.40) | 0.73 | 1.05 (0.78, 1.41) | 0.73 | 1.11 (0.82, 1.51) | 0.50 |
| 5^th^ decile (f 5.7-6.0; m 6.1-.4) | 1.23 (0.94, 1.61) | 0.13 | 1.20 (0.91, 1.58) | 0.20 | 1.19 (0.89, 1.59) | 0.24 |
| 6^th^ decile (f 6.1-6.3; m 6.5-6.8) | 1.02 (0.76, 1.37) | 0.90 | 1.03 (0.76, 1.40) | 0.84 | 0.99 (0.72, 1.37) | 0.96 |
| 7^th^ decile (f 6.4-6.8; m 6.9-7.2) | 1.15 (0.88, 1.52) | 0.30 | 1.14 (0.86, 1.51) | 0.35 | 1.11 (0.83, 1.49) | 0.48 |
| 8^th^ decile (f 6.9-7.3; m 7.3-7.8) | 1.37 (1.05, 1.78) | 0.02 | 1.31 (0.99, 1.72) | 0.06 | 1.28 (0.96, 1.71) | 0.09 |
| 9^th^ decile (f 7.4-8.2; m 7.9-8.7) | 1.36 (1.04, 1.77) | 0.02 | 1.24 (0.94, 1.63) | 0.14 | 1.25 (0.94, 1.68) | 0.13 |
| 10^th^ decile (f 8.2-10; m 8.8-10) | 1.14 (0.85, 1.54) | 0.39 | 1.03 (0.75, 1.41) | 0.86 | 1.04 (0.75, 1.44) | 0.83 |
| **Respiratory disease mortality (no. of deaths=294)** |  |  |  |  |  |  |
| 1^st^ decile (f 4.0-4.5; m 4.0-4.7) | 1.12 (0.57, 2.20) | 0.74 | 1.06 (0.53, 2.13) | 0.78 | 1.08 (0.52, 2.24) | 0.84 |
| 2^nd^ decile (f 4.5-4.8; m 4.7-5.2) | 1.19 (0.66, 2.14) | 0.56 | 1.23 (0.68, 2.23) | 0.50 | 1.30 (0.70, 2.41) | 0.41 |
| 3^rd^ decile (f 4.9-5.3; m 5.3-5.7) | Reference |  | Reference |  | Reference |  |
| 4^th^ decile (f 5.4-5.6; m 5.8-6.0) | 1.36 (0.78, 2.36) | 0.28 | 1.42 (0.80, 2.52) | 0.24 | 1.56 (0.86, 2.81) | 0.14 |
| 5^th^ decile (f 5.7-6.0; m 6.1-.4) | 1.43 (0.84, 2.46) | 0.19 | 1.52 (0.87, 2.65) | 0.14 | 1.50 (0.84, 2.70) | 0.17 |
| 6^th^ decile (f 6.1-6.3; m 6.5-6.8) | 1.41 (0.80, 2.49) | 0.24 | 1.48 (0.82, 2.65) | 0.20 | 1.62 (0.89, 2.96) | 0.12 |
| 7^th^ decile (f 6.4-6.8; m 6.9-7.2) | 1.46 (0.85, 2.52) | 0.17 | 1.55 (0.88, 2.72) | 0.12 | 1.53 (0.85, 2.76) | 0.16 |
| 8^th^ decile (f 6.9-7.3; m 7.3-7.8) | 2.07 (1.24, 3.45) | 0.005 | 2.17 (1.28, 3.69) | 0.004 | 2.27 (1.31, 3.94) | 0.003 |
| 9^th^ decile (f 7.4-8.2; m 7.9-8.7) | 1.99 (1.19, 3.34) | 0.009 | 2.25 (1.32, 3.83) | <0.001 | 2.27 (1.30, 3.96) | 0.004 |
| 10^th^ decile (f 8.2-10; m 8.8-10) | 1.83 (1.05, 3.18) | 0.03 | 2.00 (1.12, 3.56) | 0.001 | 2.11 (1.16, 3.83) | 0.01 |

^a^: adjusted for age, sex, education, occupation, smoking status, alcohol use, physical activity, self-rated health, body mass index, diabetes, triglycerides, high-density lipoprotein cholesterol, platelet count and haemoglobin.

Table S3. Association between the baseline white blood cell count within normal range (4 ×10^9^/L < WBC count < 10 ×10^9^/L) and respiratory disease mortality in the Guangzhou Biobank Cohort Study, 2003-2016, excluding deaths within 2 years

| **Decile of WBC count, ×10^9^/L (range)** | **Multivariable model ^a^**  **(n=27,668)** | | **Multivariable model in**  **non-smokers ^a^ (n=** **22,361)** | |
| --- | --- | --- | --- | --- |
|  | HR (95% CI) | *P* value | HR (95% CI) | *P* value |
| 1^st^ decile (f 4.0-4.5; m 4.0-4.7) | 1.20 (0.58, 2.50) | 0.63 | 1.13 (0.52, 2.80) | 0.82 |
| 2^nd^ decile (f 4.5-4.8; m 4.7-5.2) | 1.20 (0.64, 2.24) | 0.57 | 1.06 (0.45, 2.54) | 0.90 |
| 3^rd^ decile (f 4.9-5.3; m 5.3-5.7) | Reference |  | Reference |  |
| 4^th^ decile (f 5.4-5.6; m 5.8-6.0) | 1.51 (0.83, 2.73) | 0.18 | 1.49 (0.69, 3.31) | 0.32 |
| 5^th^ decile (f 5.7-6.0; m 6.1-.4) | 1.41 (0.79, 2.54) | 0.24 | 1.42 (0.67, 3.14) | 0.38 |
| 6^th^ decile (f 6.1-6.3; m 6.5-6.8) | 1.47 (0.80, 2.70) | 0.21 | 1.68 (0.79, 3.85) | 0.20 |
| 7^th^ decile (f 6.4-6.8; m 6.9-7.2) | 1.36 (0.75, 2.46) | 0.31 | 1.36 (0.64, 3.03) | 0.44 |
| 8^th^ decile (f 6.9-7.3; m 7.3-7.8) | 1.89 (1.08, 3.30) | 0.03 | 1.87 (0.90, 4.04) | 0.10 |
| 9^th^ decile (f 7.4-8.2; m 7.9-8.7) | 1.01 (1.15, 3.52) | 0.01 | 2.06 (1.01, 4.48) | 0.06 |
| 10^th^ decile (f 8.2-10; m 8.8-10) | 1.70 (0.92, 3.14) | 0.09 | 1.54 (0.83, 3.84) | 0.31 |

^a^: adjusted for age, sex, education, occupation, smoking status, alcohol use, physical activity, self-rated health, body mass index, diabetes, triglycerides, high-density lipoprotein cholesterol, platelet count, haemoglobin, force vital capacity (FVC), forced expiratory volume in 1 second (FEV1), and self-reported respiratory disease (COPD, chronic bronchitis, emphysema, asthma, tuberculosis, pneumonia)

Table S4. Association between the baseline white blood cell count and respiratory disease mortality in current smokers in the Guangzhou Biobank Cohort Study, 2003-2016

| **WBC count quartile, ×10^9^/L**  **(range)** | **Age, sex-adjusted results**  **(n=3040)** | | **Multivariable adjusted ^a^**  **(n=3040)** | | **Further excluding deaths**  **within 2 years ^a^ (n=3001)** | |
| --- | --- | --- | --- | --- | --- | --- |
|  | HR (95% CI) | *P* value | HR (95% CI) | *P* value | HR (95% CI) | *P* value |
| 1^st^ Quartile (5.24 or below) | 1.47 (0.56, 3.82) | 0.43 | 1.07 (0.39, 2.92) | 0.9 | 1.16 (0.41, 3.29) | 0.78 |
| 2^nd^ Quartile (5.25 – 6.20) | Reference |  | Reference |  | Reference |  |
| 3^rd^ Quartile (6.22 – 7.21) | 1.28 (0.53, 3.1) | 0.58 | 1.24 (0.49, 3.1) | 0.65 | 1.11 (0.41, 3.02) | 0.84 |
| 4^th^ Quartile (7.22 or above) | 2.11 (1.01, 4.4) | 0.05 | 2.22 (1.03, 4.79) | 0.04 | 2.36 (1.05, 5.3) | 0.04 |

a: adjusted for age, sex, education, occupation, smoking status, alcohol use, physical activity, self-rated health, body mass index, diabetes, triglycerides, high-density lipoprotein cholesterol, platelet count and haemoglobin

Table S5. Association between the baseline granulocyte count and total mortality and coronary heart disease (CHD) mortality in the Guangzhou Biobank Cohort Study, 2003-2016

| **Decile of granulocyte count, ×10^9^/L (range)** | **Age, sex-adjusted results**  **(n=29,859)** | | **Multivariable adjusted^a^**  **(n=29,859)** | | **Further excluding deaths**  **within 2 years ^a^ (n=29,607)** | |
| --- | --- | --- | --- | --- | --- | --- |
|  | HR (95% CI) | *P* value | HR (95% CI) | *P* value | HR (95% CI) | *P* value |
| **Total mortality (no. of deaths=2,959)** | | | | | | |
| 1^st^ decile (<2.40) | Reference |  | Reference |  | Reference |  |
| 2^nd^ decile (2.50-2.80) | 0.87 (0.73, 1.03) | 0.11 | 0.87 (0.72, 1.04) | 0.13 | 0.85 (0.70, 1.03) | 0.10 |
| 3^rd^ decile (2.82-3.10) | 0.79 (0.66, 0.94) | 0.01 | 0.82 (0.68, 0.98) | 0.03 | 0.82 (0.68, 1.00) | 0.05 |
| 4^th^ decile (3.13-3.40) | 0.76 (0.63, 0.9) | 0.002 | 0.8 (0.66, 0.96) | 0.02 | 0.80 (0.66, 0.97) | 0.03 |
| 5^th^ decile (3.42-3.60) | 0.8 (0.66, 0.97) | 0.02 | 0.85 (0.69, 1.04) | 0.11 | 0.86 (0.70, 1.06) | 0.17 |
| 6^th^ decile (3.64-3.90) | 0.81 (0.68, 0.97) | 0.02 | 0.86 (0.71, 1.03) | 0.09 | 0.87 (0.72, 1.06) | 0.16 |
| 7^th^ decile (3.92-4.30) | 0.96 (0.81, 1.13) | 0.6 | 0.99 (0.83, 1.17) | 0.87 | 1.00 (0.83, 1.20) | 0.99 |
| 8^th^ decile (4.35-4.70) | 1.11 (0.94, 1.31) | 0.23 | 1.13 (0.94, 1.35) | 0.18 | 1.14 (0.94, 1.37) | 0.17 |
| 9^th^ decile (4.79-5.40) | 1.19 (1.01, 1.4) | 0.04 | 1.17 (0.99, 1.39) | 0.07 | 1.15 (0.96, 1.38) | 0.13 |
| 10^th^ decile (>5.44) | 1.53 (1.31, 1.78) | <0.001 | 1.48 (1.25, 1.75) | <0.001 | 1.45 (1.21, 1.73) | <0.001 |
| **CHD mortality (no. of deaths=457)^b^** | | | | | | |
| 1^st^ decile (<2.40) | Reference |  | Reference |  | Reference |  |
| 2^nd^ decile (2.50-2.80) | 0.55 (0.30, 1.00) | 0.05 | 0.56 (0.31, 1.01) | 0.05 | 0.5 (0.27, 0.92) | 0.03 |
| 3^rd^ decile (2.82-3.10) | 0.87 (0.51, 1.49) | 0.62 | 0.83 (0.49, 1.43) | 0.51 | 0.84 (0.49, 1.44) | 0.53 |
| 4^th^ decile (3.13-3.40) | 0.76 (0.45, 1.3) | 0.32 | 0.72 (0.42, 1.26) | 0.25 | 0.73 (0.42, 1.26) | 0.26 |
| 5^th^ decile (3.42-3.60) | 1.16 (0.68, 1.96) | 0.59 | 1.08 (0.63, 1.87) | 0.77 | 1.05 (0.61, 1.83) | 0.85 |
| 6^th^ decile (3.64-3.90) | 0.76 (0.44, 1.3) | 0.31 | 0.72 (0.42, 1.25) | 0.24 | 0.73 (0.42, 1.26) | 0.26 |
| 7^th^ decile (3.92-4.30) | 1.3 (0.81, 2.08) | 0.27 | 1.19 (0.73, 1.93) | 0.5 | 1.21 (0.74, 1.96) | 0.45 |
| 8^th^ decile (4.35-4.70) | 1.65 (1.03, 2.62) | 0.04 | 1.5 (0.92, 2.43) | 0.1 | 1.41 (0.86, 2.31) | 0.17 |
| 9^th^ decile (4.79-5.40) | 1.48 (0.93, 2.36) | 0.1 | 1.37 (0.85, 2.23) | 0.2 | 1.31 (0.8, 2.14) | 0.29 |
| 10^th^ decile (>5.44) | 2.12 (1.37, 3.29) | <0.001 | 1.8 (1.12, 2.89) | 0.02 | 1.69 (1.04, 2.73) | 0.03 |

^a^: adjusted for age, sex, education, occupation, smoking status, alcohol use, physical activity, self-rated health, body mass index, diabetes, triglycerides, high-density lipoprotein cholesterol, platelet count and haemoglobin.

^b^: 1528 participant with vascular disease at baseline including coronary heart disease, stroke, angina, myocardial infarction and peripheral vascular disease were excluded.

Table S6. Association between the baseline granulocyte count and cancer and respiratory disease mortality in the Guangzhou Biobank Cohort Study, 2003-2016

| **Decile of granulocyte count,** **×10^9^/L (range)** | **Age, sex-adjusted results**  **(n=29,859)** | | **Multivariable adjusted ^a^**  **(n=29,859)** | | **Further excluding deaths**  **within 2 years ^a^ (n=29,607)** | |
| --- | --- | --- | --- | --- | --- | --- |
|  | HR (95% CI) | *P* value | HR (95% CI) | *P* value | HR (95% CI) | *P* value |
| **Cancer mortality (no. of deaths=1,143)** |  |  |  |  |  |  |
| 1^st^ decile (<2.40) | Reference |  | Reference |  | Reference |  |
| 2^nd^ decile (2.50-2.80) | 1.11 (0.85, 1.44) | 0.44 | 1.13 (0.86, 1.48) | 0.39 | 1.12 (0.84, 1.49) | 0.45 |
| 3^rd^ decile (2.82-3.10) | 0.92 (0.70, 1.21) | 0.56 | 0.95 (0.72, 1.26) | 0.73 | 0.91 (0.68, 1.23) | 0.56 |
| 4^th^ decile (3.13-3.40) | 0.67 (0.50, 0.90) | 0.01 | 0.68 (0.5, 0.93) | 0.01 | 0.67 (0.49, 0.93) | 0.02 |
| 5^th^ decile (3.42-3.60) | 0.79 (0.58, 1.08) | 0.14 | 0.82 (0.59, 1.13) | 0.23 | 0.86 (0.62, 1.20) | 0.37 |
| 6^th^ decile (3.64-3.90) | 0.93 (0.71, 1.22) | 0.59 | 0.96 (0.72, 1.27) | 0.76 | 0.93 (0.69, 1.25) | 0.62 |
| 7^th^ decile (3.92-4.30) | 0.84 (0.64, 1.1) | 0.21 | 0.85 (0.64, 1.12) | 0.25 | 0.84 (0.62, 1.13) | 0.24 |
| 8^th^ decile (4.35-4.70) | 0.81 (0.61, 1.08) | 0.15 | 0.78 (0.58, 1.06) | 0.11 | 0.79 (0.57, 1.08) | 0.15 |
| 9^th^ decile (4.79-5.40) | 1.11 (0.86, 1.44) | 0.41 | 1.06 (0.80, 1.40) | 0.69 | 1.04 (0.78, 1.40) | 0.78 |
| 10^th^ decile (>5.44) | 0.95 (0.72, 1.24) | 0.69 | 0.84 (0.63, 1.13) | 0.26 | 0.79 (0.58, 1.08) | 0.14 |
| **Respiratory disease mortality (no. of deaths=323)** |  |  |  |  |  |  |
| 1^st^ decile (<2.40) | Reference |  | Reference |  | Reference |  |
| 2^nd^ decile (2.50-2.80) | 0.93 (0.5, 1.75) | 0.83 | 0.95 (0.48, 1.86) | 0.88 | 0.78 (0.39, 1.59) | 0.5 |
| 3^rd^ decile (2.82-3.10) | 1.11 (0.6, 2.03) | 0.74 | 1.34 (0.71, 2.52) | 0.37 | 1.28 (0.67, 2.42) | 0.46 |
| 4^th^ decile (3.13-3.40) | 1.1 (0.61, 1.98) | 0.76 | 1.25 (0.66, 2.36) | 0.49 | 1.24 (0.65, 2.33) | 0.52 |
| 5^th^ decile (3.42-3.60) | 0.98 (0.5, 1.89) | 0.94 | 1.27 (0.64, 2.54) | 0.5 | 1.11 (0.54, 2.26) | 0.78 |
| 6^th^ decile (3.64-3.90) | 1.08 (0.6, 1.97) | 0.79 | 1.40 (0.75, 2.63) | 0.29 | 1.34 (0.71, 2.52) | 0.37 |
| 7^th^ decile (3.92-4.30) | 1.47 (0.84, 2.56) | 0.18 | 1.82 (1.00, 3.31) | 0.05 | 1.66 (0.90, 3.03) | 0.1 |
| 8^th^ decile (4.35-4.70) | 1.57 (0.89, 2.76) | 0.12 | 1.97 (1.07, 3.62) | 0.03 | 1.89 (1.02, 3.48) | 0.04 |
| 9^th^ decile (4.79-5.40) | 1.48 (0.85, 2.58) | 0.17 | 1.81 (0.99, 3.30) | 0.05 | 1.60 (0.87, 2.94) | 0.13 |
| 10^th^ decile (>5.44) | 2.44 (1.44, 4.11) | <0.001 | 2.89 (1.62, 5.14) | <0.001 | 2.66 (1.49, 4.75) | <0.001 |

^a^: adjusted for age, sex, education, occupation, smoking status, alcohol use, physical activity, self-rated health, body mass index, diabetes, triglycerides, high-density lipoprotein cholesterol, platelet count and haemoglobin.

Table S7. Association between the baseline lymphocyte count and total mortality and coronary heart disease (CHD) mortality in the Guangzhou Biobank Cohort Study, 2003-2016

| **Decile of lymphocyte count, ×10^9^/L (range)** | **Age, sex-adjusted results**  **(n=29,859)** | | **Multivariable adjusted^a^**  **(n=29,859)** | | **Further excluding deaths**  **within 2 years ^a^ (n=29,607)** | |
| --- | --- | --- | --- | --- | --- | --- |
|  | HR (95% CI) | *P* value | HR (95% CI) | *P* value | HR (95% CI) | *P* value |
| **Total mortality (no. of deaths=2,976)** | | | | | | |
| 1^st^ decile (<1.40) | Reference |  | Reference |  | Reference |  |
| 2^nd^ decile (1.42-1.60) | 0.89 (0.78, 1.03) | 0.12 | 0.89 (0.77, 1.03) | 0.12 | 0.94 (0.81, 1.09) | 0.43 |
| 3^rd^ decile (1.61-1.80) | 0.89 (0.78, 1.02) | 0.1 | 0.88 (0.76, 1.01) | 0.06 | 0.91 (0.79, 1.06) | 0.22 |
| 4^th^ decile (1.81-1.90) | 0.87 (0.74, 1.03) | 0.1 | 0.82 (0.7, 0.98) | 0.03 | 0.86 (0.72, 1.02) | 0.09 |
| 5^th^ decile (1.93-2.10) | 0.78 (0.68, 0.9) | <0.001 | 0.76 (0.66, 0.87) | <0.001 | 0.75 (0.65, 0.88) | <0.001 |
| 6^th^ decile (2.12-2.20) | 0.79 (0.66, 0.94) | 0.01 | 0.79 (0.65, 0.94) | 0.01 | 0.82 (0.68, 0.99) | 0.04 |
| 7^th^ decile (2.21-2.40) | 0.86 (0.74, 0.99) | 0.04 | 0.82 (0.7, 0.95) | 0.01 | 0.84 (0.71, 0.98) | 0.03 |
| 8^th^ decile (2.42-2.60) | 0.82 (0.7, 0.96) | 0.02 | 0.77 (0.65, 0.91) | <0.001 | 0.81 (0.68, 0.97) | 0.02 |
| 9^th^ decile (2.62-2.90) | 0.79 (0.67, 0.93) | 0.01 | 0.73 (0.61, 0.86) | <0.001 | 0.73 (0.61, 0.88) | <0.001 |
| 10^th^ decile (>2.91) | 0.9 (0.77, 1.06) | 0.21 | 0.82 (0.69, 0.97) | 0.02 | 0.86 (0.72, 1.02) | 0.09 |
| **CHD mortality (no. of deaths=457)^b^** | | | | | | |
| 1^st^ decile (<1.40) | Reference |  | Reference |  | Reference |  |
| 2^nd^ decile (1.42-1.60) | 1.09 (0.75, 1.6) | 0.64 | 1.04 (0.7, 1.54) | 0.84 | 1.08 (0.72, 1.61) | 0.72 |
| 3^rd^ decile (1.61-1.80) | 0.86 (0.58, 1.26) | 0.44 | 0.84 (0.56, 1.24) | 0.38 | 0.88 (0.59, 1.31) | 0.52 |
| 4^th^ decile (1.81-1.90) | 0.98 (0.63, 1.54) | 0.94 | 0.86 (0.54, 1.37) | 0.53 | 0.79 (0.48, 1.29) | 0.35 |
| 5^th^ decile (1.93-2.10) | 0.81 (0.55, 1.19) | 0.27 | 0.75 (0.5, 1.12) | 0.16 | 0.75 (0.5, 1.13) | 0.17 |
| 6^th^ decile (2.12-2.20) | 0.9 (0.56, 1.46) | 0.67 | 0.87 (0.53, 1.42) | 0.57 | 0.85 (0.51, 1.41) | 0.53 |
| 7^th^ decile (2.21-2.40) | 0.91 (0.61, 1.37) | 0.65 | 0.82 (0.53, 1.25) | 0.35 | 0.85 (0.55, 1.32) | 0.47 |
| 8^th^ decile (2.42-2.60) | 0.9 (0.57, 1.4) | 0.63 | 0.79 (0.49, 1.25) | 0.31 | 0.84 (0.53, 1.34) | 0.47 |
| 9^th^ decile (2.62-2.90) | 1.14 (0.75, 1.73) | 0.54 | 0.91 (0.58, 1.41) | 0.66 | 0.89 (0.56, 1.42) | 0.64 |
| 10^th^ decile (>2.91) | 0.71 (0.44, 1.16) | 0.17 | 0.58 (0.35, 0.98) | 0.04 | 0.63 (0.37, 1.06) | 0.08 |

^a^: adjusted for age, sex, education, occupation, smoking status, alcohol use, physical activity, self-rated health, body mass index, diabetes, triglycerides, high-density lipoprotein cholesterol, platelet count and haemoglobin.

^b^: 1528 participant with vascular disease at baseline including coronary heart disease, stroke, angina, myocardial infarction and peripheral vascular disease were excluded.

Table S8. Association between the baseline lymphocyte count and cancer and respiratory disease mortality in the Guangzhou Biobank Cohort Study, 2003-2016

| **Decile of lymphocyte count, ×10^9^/L (range)** | **Age, sex-adjusted results**  **(n=29,859)** | | **Multivariable adjusted ^a^**  **(n=29,859)** | | **Further excluding deaths**  **within 2 years ^a^ (n=29,607)** | |
| --- | --- | --- | --- | --- | --- | --- |
|  | HR (95% CI) | *P* value | HR (95% CI) | *P* value | HR (95% CI) | *P* value |
| **Cancer mortality (no. of deaths=1,143)** |  |  |  |  |  |  |
| 1^st^ decile (<1.40) | Reference |  | Reference |  | Reference |  |
| 2^nd^ decile (1.42-1.60) | 0.94 (0.75, 1.19) | 0.61 | 0.94 (0.74, 1.19) | 0.6 | 1.04 (0.81, 1.34) | 0.73 |
| 3^rd^ decile (1.61-1.80) | 0.83 (0.66, 1.04) | 0.1 | 0.78 (0.62, 0.99) | 0.04 | 0.84 (0.66, 1.08) | 0.18 |
| 4^th^ decile (1.81-1.90) | 0.74 (0.56, 0.98) | 0.04 | 0.68 (0.5, 0.91) | 0.01 | 0.73 (0.54, 1.00) | 0.05 |
| 5^th^ decile (1.93-2.10) | 0.82 (0.65, 1.03) | 0.08 | 0.8 (0.64, 1.01) | 0.06 | 0.80 (0.62, 1.02) | 0.08 |
| 6^th^ decile (2.12-2.20) | 0.79 (0.59, 1.05) | 0.11 | 0.78 (0.58, 1.05) | 0.1 | 0.87 (0.63, 1.18) | 0.37 |
| 7^th^ decile (2.21-2.40) | 0.77 (0.6, 0.99) | 0.04 | 0.72 (0.56, 0.94) | 0.01 | 0.76 (0.58, 1.00) | 0.05 |
| 8^th^ decile (2.42-2.60) | 0.9 (0.69, 1.16) | 0.4 | 0.82 (0.63, 1.07) | 0.15 | 0.88 (0.66, 1.16) | 0.36 |
| 9^th^ decile (2.62-2.90) | 0.66 (0.5, 0.88) | <0.001 | 0.61 (0.45, 0.82) | <0.001 | 0.63 (0.46, 0.87) | <0.001 |
| 10^th^ decile (>2.91) | 0.97 (0.75, 1.25) | 0.79 | 0.88 (0.67, 1.16) | 0.37 | 0.96 (0.72, 1.27) | 0.76 |
| **Respiratory disease mortality (no. of deaths=323)** |  |  |  |  |  |  |
| 1^st^ decile (<1.40) | Reference |  | Reference |  | Reference |  |
| 2^nd^ decile (1.42-1.60) | 0.83 (0.53, 1.31) | 0.43 | 0.86 (0.54, 1.36) | 0.51 | 0.87 (0.55, 1.39) | 0.57 |
| 3^rd^ decile (1.61-1.80) | 1.34 (0.91, 1.98) | 0.14 | 1.40 (0.94, 2.08) | 0.1 | 1.27 (0.84, 1.91) | 0.26 |
| 4^th^ decile (1.81-1.90) | 1.16 (0.72, 1.88) | 0.55 | 1.23 (0.75, 2.00) | 0.41 | 1.05 (0.63, 1.76) | 0.85 |
| 5^th^ decile (1.93-2.10) | 1.01 (0.67, 1.52) | 0.98 | 1.00 (0.65, 1.54) | 0.99 | 0.99 (0.64, 1.52) | 0.95 |
| 6^th^ decile (2.12-2.20) | 0.78 (0.44, 1.4) | 0.41 | 0.82 (0.45, 1.52) | 0.54 | 0.83 (0.45, 1.53) | 0.54 |
| 7^th^ decile (2.21-2.40) | 1.02 (0.65, 1.61) | 0.92 | 0.99 (0.61, 1.60) | 0.95 | 0.92 (0.56, 1.51) | 0.75 |
| 8^th^ decile (2.42-2.60) | 0.97 (0.59, 1.6) | 0.91 | 1.09 (0.65, 1.81) | 0.75 | 1.04 (0.62, 1.75) | 0.88 |
| 9^th^ decile (2.62-2.90) | 0.88 (0.52, 1.48) | 0.62 | 0.99 (0.58, 1.69) | 0.97 | 0.89 (0.51, 1.56) | 0.69 |
| 10^th^ decile (>2.91) | 1.05 (0.64, 1.73) | 0.85 | 1.21 (0.72, 2.02) | 0.48 | 1.11 (0.65, 1.89) | 0.71 |

^a^: adjusted for age, sex, education, occupation, smoking status, alcohol use, physical activity, self-rated health, body mass index, diabetes, triglycerides, high-density lipoprotein cholesterol, platelet count and haemoglobin.
